# Bubble-Free Frontal Polymerization of Acrylates via Redox-Initiated Free Radical Polymerization

**DOI:** 10.3390/polym16192830

**Published:** 2024-10-07

**Authors:** Morteza Ziaee, Mostafa Yourdkhani

**Affiliations:** 1 Department of Mechanical Engineering, Colorado State University, Fort Collins, CO 80523, USA; 2 School of Manufacturing Systems & Networks, Ira A. Fulton Schools of Engineering, Arizona State University, Mesa, AZ 85212, USA

**Keywords:** frontal polymerization, free-radical frontal polymerization, peroxide initiator, redox initiator, acrylates, bubble formation

## Abstract

Thermal frontal polymerization (FP) of acrylate monomers mixed with conventional peroxide initiators leads to significant bubble formation at the polymerizing front, limiting their practical applications. Redox initiators present a promising alternative to peroxide initiators, as they prevent the formation of gaseous byproducts during initiator decomposition and lower the front temperature, thereby enabling bubble-free FP. In this study, we investigate the FP of acrylate monomers of varying functionalities, including methyl methacrylate (MMA), 1,6-hexanediol diacrylate (HDDA), and trimethylolpropane triacrylate (TMPTA), using *N*,*N*-dimethylaniline/benzoyl peroxide (DMA/BPO) redox couple at room temperature and compare their front behavior, pot life, and bubble formation with those of same resin systems mixed with a conventional peroxide initiator, Luperox 231. The use of redox couples in FP of acrylates shows promise for rapid, energy-efficient manufacturing of polyacrylates and can enable new applications such as 3D printing and composite manufacturing.

## 1. Introduction

Frontal polymerization (FP) is a promising curing strategy that can drastically reduce the energy consumption and manufacturing time of polymeric materials, offering a sustainable solution for large-scale production of polymers and their composites [1,2,3,4]. FP is a polymerization approach, in which a resin filled with a thermally latent initiator cures on demand at ambient conditions through activation via a hot trigger. Once FP is started by activating the initiator, a self-sustaining reaction wave is produced, which propagates through the monomers by coupling thermal diffusion with the Arrhenius kinetic of the exothermic reaction to convert unreacted monomers to a solid polymer [5,6].

Free-radical polymerization is one of the most common polymerization schemes studied for thermal FP of polymers, especially for acrylate monomers. This polymerization mechanism involves thermal-induced decomposition of latent initiators into reactive free radical species that quickly react with monomer to form polymer chains. An early demonstration of free-radical FP was carried out on methyl methacrylate (MMA) using benzoyl peroxide (BPO) as a thermal latent initiator in a closed reaction vessel under a high pressure (3500 atm) [7]. This high pressure was used to promote the rate of polymerization and eliminate convective instabilities in front propagation; however, such extreme conditions are not easily adaptable to various manufacturing processes. Compared to methacrylate monomers, free-radical FP of multifunctional acrylates can be readily conducted at the ambient pressure, owing to their higher reactivity [8,9,10]. Extensive research has been carried out using multifunctional monomer systems for various applications, including on-demand curing of adhesives and coatings for wood substrates [11], rapid fabrication of anisotropic structural foams [12], manufacturing of microfluidic endoskeletons [13], facile synthesis of hydrogels [14,15], creation of functionally gradient materials [16], and rapid consolidation of stones [17,18].

The most common types of thermal initiators used for FP of multifunctional acrylates are peroxide and nitrile initiators, including BPO, *tert*-butylperoxide (*t*-BPO), cumene hydroperoxide (CHP), 1,1-Di-(*tert*-butylperoxy)-3,3,5-trimethylcyclohexane (Luperox 231), and 2,2-azobis(isobutyronitrile) (AIBN) [10,16,19,20,21]. However, FP using the above initiators has several shortcomings that limit its practical applications. For example, nitrile and peroxide initiators generate volatile components upon decomposition of the initiator that can create a large volume of voids and substantially reduce the mechanical performance of the produced polymers [22]. In addition, FP of multifunctional acrylates is highly exothermic and lacks sufficient control over front temperature. FP of diacrylate and triacrylate monomers using the above initiators typically results in front temperatures exceeding 300 °C, leading to the boiling of monomers and collapse of the propagating front [9]. Polymerization at such elevated temperatures may result in degradation of the cured polymer or even the thermal decomposition of the initiator, resulting in low conversions [23].

Several approaches have been explored to address the problems associated with the FP of acrylate monomers by reducing the front temperature. One approach is FP in the presence of peracrylates that decompose upon copolymerization with acrylate monomers, allowing FP to occur at lower temperatures [24]. This method can reduce bubble formation but adversely affects the stability of the polymerization front. Additionally, inert fillers or nonvolatile deep eutectic solvents have been utilized to absorb the heat of polymerization and reduce front temperature [25,26]. In another approach, frontal copolymerization of acrylate monomers with thiol-acrylate systems was performed to lower the enthalpy of polymerization [27]. The addition of thiol-ene monomers can decrease the front temperature and make the produced polymer more flexible, but it significantly shortens the pot life of the resin.

Alternatively, redox initiators have been shown to be effective for free-radical FP under a controlled reaction condition. The first redox FP was demonstrated using *N*,*N*-dimethylaniline/benzoyl peroxide (DMA/BPO) and ammonium persulfate (APS)/*N*,*N*,*N*′,*N*′-tetramethylethylenediamine (TMEDA) couples for bubble-free synthesis of hydrogel systems [28]. Subsequently, a new redox pairing consisting of APS and carbon dots was used to conduct FP of hydrogels, resulting in a considerable reduction in front temperature [29]. In another study, iodonium salts and amines were used to demonstrate redox-based FP of polyacrylates, where the front velocity could be controlled by adjusting the ratio of oxidant to reductant; however, void formation still remains as a major challenge in this method [30]. In a follow-up study, the amine reductant was replaced with a phosphine compound to avoid bubble formation during the FP of polyacrylates, but this method suffered from a short pot life [31].

Given the promising results for bubble-free FP of hydrogel systems using the DMA/BPO redox couple, here we present the use of this redox couple for FP of acrylate monomers at room temperature. Initially, we investigate the FP of 1,6-hexanediol diacrylate (HDDA) using a conventional peroxide initiator, Luperox 231, as a baseline system. Then, we replace the peroxide initiator with the DMA/BPO redox couple to address the limitations associated with the peroxide initiator and examine the impact of reductant/oxidant ratio on front behavior and pot life. In the next step, the HDDA monomer is replaced with monoacrylate and triacrylate monomers to study their FP using both peroxide and redox initiators and to reveal the effect of monomer functionality on front behavior.

## 2. Materials and Methods

### 2.1. Materials

**Figure 1:**
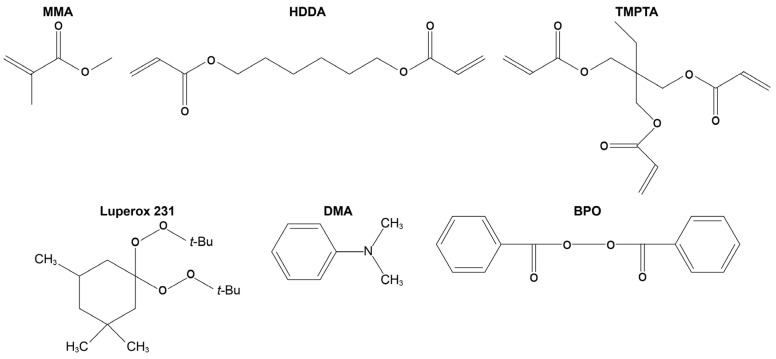
Chemical structures of reagents used in this study.

Methyl methacrylate (MMA) was used as a monofunctional monomer and purchased from TCI America. HDDA and trimethylolpropane triacrylate (TMPTA) were used as difunctional and trifunctional monomers, respectively, acquired from Alfa Aesar. The redox couple consisted of BPO and DMA, sourced from Thermo Scientific Chemicals. Luperox 231 was used as a peroxide initiator and obtained from Sigma-Aldrich. All chemicals are commercially available and used as received without further purification. The chemical structures of the monomers and the initiators used in this study are shown in Figure 1.

### 2.2. Preparation of Reactant Solutions

In all experiments, 12 g of monomers were weighed out into a 100 mL disposable cup. For preparation of FP solutions containing the peroxide initiator, 0.4 phr (parts per hundred resin) Luperox 231 was mixed with the resins for 2 min using a planetary centrifugal mixer (AR-100, Thinky, Laguna Hills, CA, USA). For FP experiments using the redox initiator, two different amounts of BPO (0.2 or 0.4 phr) were measured and mixed with the resins using the centrifugal mixer for 2 min. After adding BPO, various molar ratios of DMA with respect to BPO (i.e., 0, 4, 8, 16 and 32 mol/mol) was pipetted into the prepared mixtures and mixed via centrifugation for 1 min. The resulting mixtures were then transferred to a 10 mL glass test tube (inner diameter = 15 mm), and the FP reaction was triggered by touching the surface of test tubes, at the top of the resin solution, using a hot soldering iron.

### 2.3. Measurement of Front Properties

**Figure 2:**
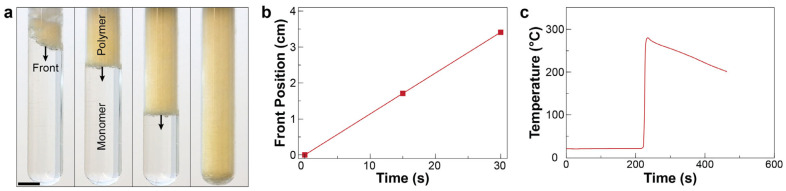
Measurement of front properties. (**a**) Sequential images captured from FP of HDDA monomer mixed with 0.4 phr Luperox 231. Scale bar represents 1 cm. (**b**) Front position as a function of time. (**c**) Temperature profile of the resin collected using a thermocouple during FP.

Once FP initiates, the heat source is removed, and front propagation is monitored using a digital camera (Figure 2a). The front velocity was calculated by plotting the front position as a function of time and finding the slope of the best fit straight line (Figure 2b). Temperature profile of resin during FP experiments is continuously measured using a K-type thermocouple connected to a thermocouple reader (Phidgets Inc., model 1048) and recorded by a custom LabVIEW code (National Instrument) at 3 Hz. The maximum temperature obtained from thermocouple was identified as a front temperature (Figure 2c). Activation time was determined as the time required for the hot trigger to initiate the self-propagating front. All experiments were carried out two times.

### 2.4. Pot Life Measurememt

To evaluate the pot life of acrylate monomer solutions in the presence of Luperox 231 and BPO initiators, individual samples were prepared, transferred into glass test tubes, and stored at room temperature (21 °C). After 24 h, samples were examined to determine whether they could still undergo FP. For the redox-FP systems containing various concentrations of DMA (in presence of BPO), samples were monitored continuously to visually identify the onset of spontaneous polymerization.

### 2.5. Evaluation of Void Formation

Following the FP experiments, the samples were removed from glass test tubes and cut using a precision saw. The cross-section of cut samples were then imaged via a digital microscope (VHX-6000, Keyence) to visually assess the extent of void formation.

## 3. Results and Discussion

### 3.1. Peroxide-Initiated Frontal Polymerization

**Figure 3:**
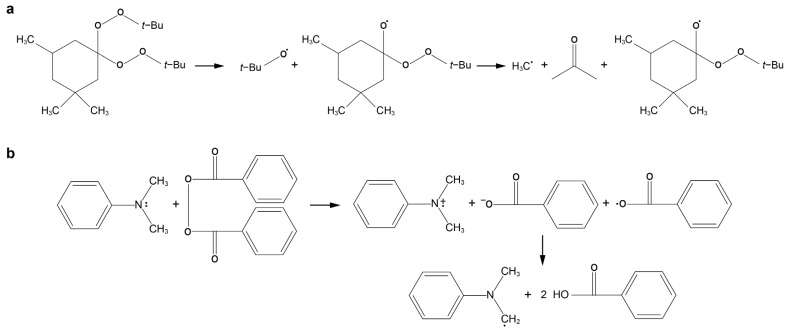
Mechanism of radical formation in (**a**) Luperox 231, and (**b**) DMA/BPO redox initiator. Reproduced with permission from [28].

Luperox 231 is a common thermal initiator for free-radical FP of various acrylates, due to its high stability at room temperature, high solubility in organic media, and relatively low gas production per radical upon decomposition compared to most peroxides and nitriles [9,21,32]. Given these attributes, we initially investigated the FP of diacrylate monomers using Luperox 231. Figure 2a shows sequential snapshots captured from front propagation in the HDDA monomer using 0.4 phr Luperox 231 as the FP initiator. FP experiments reveal significant bubble formation in the test tube, primarily caused by the decomposition of the initiator. Upon the decomposition of Luperox 231, volatile compounds, including acetone, are produced (Figure 3a) that lead to the generation of bubbles of various sizes in the sample [33,34]. The presence of small bubbles scatters light, making the material opaque.

Another source of bubble formation during the FP of diacrylate monomers is attributed to the highly exothermic nature of the reaction. In thermal FP, the increase in temperature, caused by the exothermic heat of polymerization, accelerates the reaction rate and helps the front propagate faster; however, this can induce bubble nucleation via boiling of monomers at the reaction front. Measurements of front behavior for the diacrylate monomer reveals that the front propagates at a rate of up to 7.02 cm/min, which is relatively fast compared to other frontally curable monomer systems; however, its front temperature reaches up to 282.3 °C, which can cause bubble nucleation.

### 3.2. Redox-Initiated Frontal Polymerization

**Figure 4:**
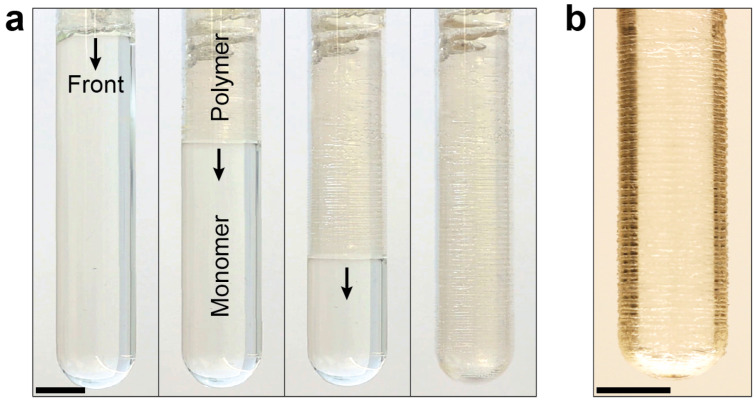
Frontal polymerization of the HDDA monomer in the presence of the redox initiator (0.2 phr BPO and DMA/BPO molar ratio = 32). (**a**) Images of front propagation. The arrows indicate the position and direction of reaction front; (**b**) Periodic pattern formed on the surface of the resulting polymeric part. Scale bars in these images represent 1 cm.

Previous studies have shown that redox initiators can be used to suppress bubble formation in free-radical FP, which is not possible by Luperox 231 alone. Our redox initiator contains the oxidant BPO and the reductant DMA, which enables FP to proceed through oxidation-reduction reaction activation mechanism. The initiation mechanism in DMA/BPO system involves an electron transfer from the nitrogen of aniline to the peroxide, resulting in the dissociation of the oxygen-oxygen bond and the generation of free radicals (Figure 3b) [28,35]. Unlike Luperox 231, the use of the redox initiator prevents bubble formation in the FP of the HDDA monomer by eliminating the production of volatile components (Figure 4a). It should be noted that the formation of some bubbles at the beginning of the frontal reaction is caused by the overheating of the monomer solution by soldering iron and unnecessary high temperature of the material at the top of the test tube (Figure 4a). However, as the front propagates toward the bottom of the test tube, no visible bubbles are formed, allowing bubble-free propagation of FP. Interestingly, FP in HDDA monomers initiated by the redox process generates periodic surface patterns on the product (Figure 4b), a phenomenon observed in FP of polymers and attributed to instabilities in the propagation of the reaction front [36].

### 3.3. Effect of DMA/BPO Ratio

**Figure 5:**
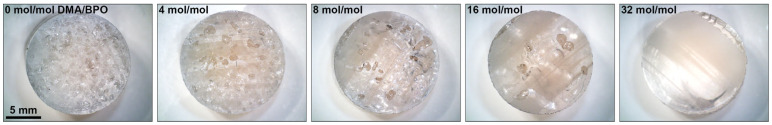
Images from the cross-section of frontally cured HDDA monomer using the redox initiator. For all samples, the BPO concentration is fixed at 0.2 phr but the DMA/BPO molar ratio varies from 0 to 32.

The efficiency of a redox system depends on the oxidant (BPO) and reductant (DMA) ratio, affecting the amount of bubble formation and front behavior. We carried out a series of FP experiments using HDDA as the frontally polymerizable monomer and various DMA/BPO molar ratios to elucidate the effect of the composition of the redox system on bubble formation, front velocity, front temperature, and activation time. Experiments were conducted using a fixed concentration of oxidant (i.e., 0.2 phr of BPO), while changing the DMA/BPO ratio from 0 to 32 mol/mol. Increasing the concentration of the DMA concentration was found to suppress bubble formation. Figure 5 shows images from the cross-section of polymer samples frontally cured in test tubes using various concentrations of DMA. These cross-sectional views indicate that a relatively clearer polymer and smoother surface can be obtained by increasing the concentration of DMA, implying less bubble formation in the cured polymer.

**Figure 6:**
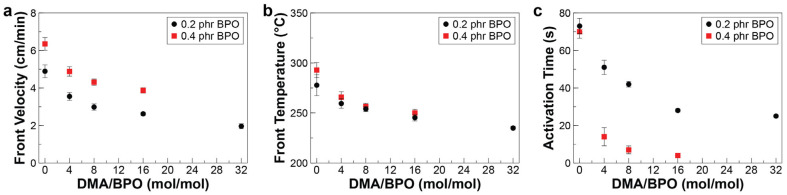
Effect of DMA/BPO ratio on frontal behavior of the HDDA diacrylate system. (**a**) Front velocity, (**b**) Front temperature, and (**c**) Activation time.

Interestingly, bubble formation is completely prevented for a DMA/BPO molar ratio of 32. The reason for this observation is that at low DMA concentrations, FP activation is mostly derived from the decomposition of the peroxide initiator, leading to the liberation of carbon dioxide. However, high DMA concentrations promote the formation of free radical species via an oxidation-reduction initiation mechanism and suppress the direct decomposition of BPO, which would otherwise produce carbon dioxide and potentially lead to bubble formation. In addition to reducing volatile byproducts, our experiments with the FP of diacrylate monomer demonstrates that the front propagates slower at a higher DMA/BPO ratio, which is related to the inhibiting effect of excess DMA on the rate of polymerization (Figure 6a). It is observed that the front velocity decreases from 4.89 to 1.96 cm/min when the DMA/BPO molar ratio is increased from 0 to 32 mol/mol, while keeping the BPO concentration constant at 0.2 phr. Reduction in front velocity prolongs the duration of heat loss through boundaries, which can consequently reduce the front temperature. Our measurements confirm that the front temperature monotonically decreases from 277.7 °C to 234.9 °C while increasing the DMA/BPO molar ratio, allowing to perform FP under milder reaction conditions (Figure 6b).

Another advantage of the redox initiator systems is their lower activation energy compared to peroxide initiators. The results of activation time measurements, defined as the time required to apply the hot trigger to the formation of self-propagating front, are shown in Figure 6c. The activation time of FP reaction in the diacrylate monomer continuously decreases when using a higher DMA concentration. A high DMA concentration enhances the rate of free-radical generation, thereby accelerating the initiation of the polymerization process [37,38].

In addition to the DMA/BPO ratio, the FP reaction is significantly influenced by the BPO concentration. Our experiments reveal that no FP occurs at any DMA/BPO ratio when the BPO concentration is below 0.2 phr, indicating the minimum peroxide concentration required to trigger FP is 0.2 phr. At low peroxide concentrations, the rate of radical production is too low to sustain the traveling front. An increase in the BPO concentration from 0.2 to 0.4 phr enhances resin reactivity, resulting in a higher front velocity, increased front temperature, and faster activation of the FP reaction (Figure 6). At a BPO concentration of 0.4 phr, the resin reactivity is so high that bulk polymerization occurs instantaneously when the DMA/BPO ratio exceeds 16.

### 3.4. Pot Life Studies

An important criterion for an FP system is that it should be stable at room temperature and have sufficient working time for use in various applications and manufacturing processes. Table 1 compares the pot life of the diacrylate resin systems in the presence of Luperox 231 and DMA/BPO redox initiators. Pot life was determined by adding the initiator to the monomer, storing the resin solution at room temperature, and observing whether the resin could still undergo FP at different time intervals [39]. A key advantage of using Luperox 231 is that it has extended pot life over 24 h. In contrast, the redox initiator has a considerably shorter pot life, due to its lower activation energy. We observed that the FP solutions prepared using either 0.2 or 0.4 phr BPO, in the absence of DMA, have a pot life of at least 24 h. However, increasing the DMA/BPO ratio at a fixed BPO concentration significantly reduces the pot life (Table 1), causing the resin to spontaneously polymerize at room temperature, due to increased initiation rate of the polymerization at higher DMA concentrations. Similarly, the pot life of resins decreases when a higher BPO concentration (0.4 phr) is used at a fixed DMA/BPO ratio. For the resin formulation without bubble formation (i.e., BPO = 0.2 phr, DMA/BPO = 32), the pot life is approximately 0.25 h. While this resin formulation results in a workable but relatively short pot life, further studies are needed to explore alternative redox initiator systems with higher activation energies compared to the DMA/BPO system to develop FP resin systems with an extended pot life.

### 3.5. Effect of Acrylate Functionality on Frontal Behavior

**Figure 7:**
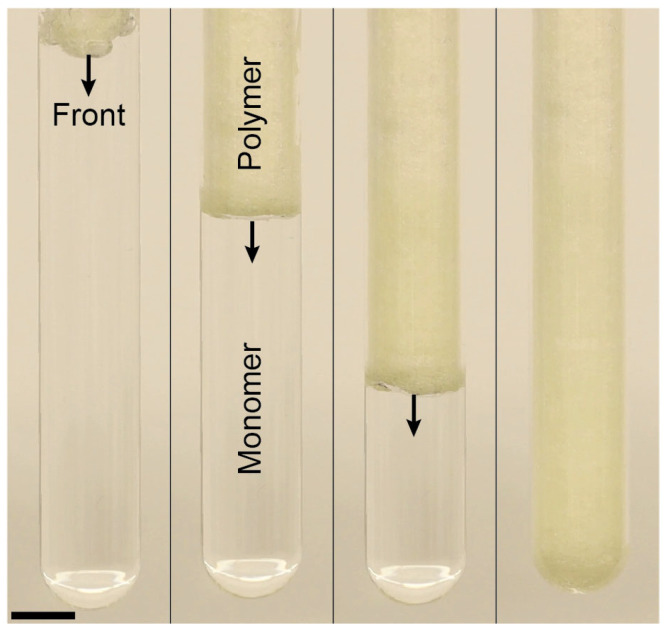
Propagation of reaction front during the FP of TMPTA triacrylate monomer in the presence of 0.4 phr Luperox 231. Scale bar represents 1 cm.

FP of monomer systems with various monomer functionality can offer rapid manufacturing of thermoplastic and thermoset polymers with controlled mechanical properties. For example, FP of monofunctional monomers can facilitate the rapid production of recyclable thermoplastic polymers, while FP of multifunctional monomers yields thermoset polymers with enhanced thermomechanical properties. Motivated by this potential, a series of FP experiments were also carried out on methyl methacrylate (MMA) and trimethylolpropane triacrylate (TMPTA) monomers using Luperox and DMA/BPO redox initiators. For samples containing the Luperox initiator, FP solutions were prepared by mixing MMA and TMPTA monomers with 0.4 phr of Luperox 231, whereas for the redox initiator systems, the monomers were mixed with either 0.2 or 0.4 phr of BPO and various DMA concentrations (4, 8, 16, and 32 mol/mol with respect to BPO). These experiments were conducted to compare the frontal behavior of these resin systems with previously measured diacrylate systems. Experiments performed using the MMA monomer reveal that this monofunctional monomer cannot support FP reaction at ambient condition, either with Luperox 231 or redox couple, due to its low intrinsic reactivity. While MMA cannot be frontally polymerized, TMPTA is highly reactive towards thermal FP reaction (Figure 7a). Frontal studies on TMPTA mixed with the Luperox 231 initiator indicate that higher front velocity (11.42 cm/min) and front temperature (319.53 °C) can be obtained compared with the diacrylate monomer, due to the higher concentration of acrylate groups in TMPTA. However, similar to the FP of the diacrylate monomer, a considerable amount of bubbles are formed during the FP of the triacrylate monomer (Figure 7). Frontal studies were also carried out on the triacrylate monomer mixed with the DMA/BPO complex; however, it was found that the triacrylate monomer system is highly unstable and immediately polymerizes upon the addition of the redox couple at any BPO concentration and DMA/BPO ratios. Although these results indicate that triacrylate monomer is highly unstable in the presence of the DMA/BPO redox couple, this problem could potentially be addressed in future studies by encapsulating one component of the redox initiator, isolating it from the other component, and releasing it on demand for immediate reaction using the thermal energy for the frontal reaction.

## 4. Conclusions

In this study, FP of acrylate monomers in the presence of peroxide and redox initiators was studied. The use of Luperox 231, a commonly used peroxide initiator for free-radical FP, results in a stable FP under ambient condition, yielding high front temperatures and rapid front propagation. However, this initiator also leads to significant bubble formation, which is undesirable for many practical applications. Alternatively, redox-initiator systems, such as the DMA/BPO system, show promise in reducing bubble formation. Our experimental studies indicate that bubble formation and front behavior can be effectively controlled by adjusting the reductant concentration. While the DMA/BPO system facilitates FP under milder conditions with substantially reduced bubble formation, its use is limited by a relatively shorter pot life. Future research should explore alternative redox systems to address this limitation, with the potential to extend their applicability to other free-radical FP systems. In addition to the choice of initiator, FP behavior is highly dependent on the reactivity of the resin, with increased monomer functionality significantly enhancing system reactivity. Our experiments with MMA (monofunctional) and TMPTA (trifunctional) monomers did not achieve successful FP under ambient conditions with the DMA/BPO redox initiator. Specifically, the former (MMA) did not support FP, while the latter (TMPTA) underwent spontaneous polymerization upon initiator addition. Extending FP to such monomer systems presents intriguing possibilities for novel applications in new manufacturing processes.

## Figures and Tables

**Table 1:** Pot life of HDDA diacrylate resin in presence of Luperox 231 and redox initiator.

Initiator	Pot Life (h)		
Luperox 231	>24		
Redox	DMA/BPO (mol/mol)	BPO (0.2 phr)	BPO (0.4 phr)
	0	>24	>24
	4	5	2
	8	2	1
	16	1	0.1
	32	0.25	0

## Data Availability

The original contributions presented in the study are included in the article, further inquiries can be directed to the corresponding author.
